# An Autopsy Case of Bronchiolitis Obliterans Associated With Oral Lichen Planus and Non-Hodgkin Lymphoma

**DOI:** 10.7759/cureus.81048

**Published:** 2025-03-23

**Authors:** Kenji Kurashina, Satoshi Hokari, Takeshi Koizumi, Satoshi Shibata, Katsuhiro Tomiyama, Hideki Hashidate, Hiroki Tsukada, Toshiaki Kikuchi

**Affiliations:** 1 Department of Respiratory Medicine and Infectious Diseases, Niigata University Graduate School of Medical and Dental Sciences, Niigata, JPN; 2 Department of Internal Medicine, Sassa General Hospital, Nishitokyo, JPN; 3 Department of General Medicine, Saiseikai Niigata Kenoh Kikan Hospital, Sanjo, JPN; 4 Department of Dermatology, Niigata City General Hospital, Niigata, JPN; 5 Department of Pathology, Niigata City General Hospital, Niigata, JPN; 6 Department of Respiratory Medicine, Niigata City General Hospital, Niigata, JPN; 7 Department of Infection Control, Jikei University Kashiwa Hospital, Kashiwa, JPN

**Keywords:** bronchiolitis obliterans, chronic respiratory failure, lichen planus, non-hodgkin lymphoma, obstructive ventilatory defect

## Abstract

We report the autopsy case of a 66-year-old woman with indolent B-cell non-Hodgkin lymphoma (NHL) who underwent repeated chemotherapy for 18 years. Two and a half years ago, she developed oral lichen planus and began corticosteroid treatment. Lichen planus recurred repeatedly with corticosteroid reduction. Obstructive ventilation impairment and respiratory failure had progressed two years ago. She was admitted to the hospital due to cytomegalovirus infection and died of respiratory failure. An autopsy revealed bronchiolitis obliterans, oral lichen planus, and recurrence of pre-existing NHL. This was a rare case of bronchiolitis obliterans with oral lichen planus and NHL.

## Introduction

Bronchiolitis obliterans (BO) is a rare but fatal respiratory disease that manifests as inflammatory and fibrotic changes in small membranous bronchioles, leading to airway lumen occlusion [1]. BO has been reported to be associated with transplantation, collagen diseases, infections, neoplasms, inhalation of toxic gas, and the ingestion of toxic substances [2]. Because a definitive diagnosis by lung biopsy is invasive, bronchiolitis obliterans syndrome (BOS) is defined for early diagnosis of chronic lung allograft dysfunction after lung transplantation or graft-versus-host disease after hematopoietic stem cell transplant: obstruction on spirometry and persistent decrease in forced expiratory volume in one second [3]. Lichen planus (LP) is a disease of unknown etiology that is thought to be caused by a T-cell autoimmune reaction against epidermal basal keratinocytes [4]. Herein, we report a rare case of BO associated with LP that resulted in death from an infectious disease during treatment for non-Hodgkin lymphoma (NHL).

## Case presentation

Our patient was a 66-year-old woman who had received chemotherapy for indolent B-cell NHL for 18 years. The chemotherapy regimen consisted of methotrexate, doxorubicin, cyclophosphamide, vincristine, prednisone, bleomycin, pirarubicin, etoposide, and rituximab. During chemotherapy, the patient developed cytomegalovirus (CMV) infection and de novo hepatitis B, which improved with ganciclovir and lamivudine plus adefovir combination therapy.

**Figure 1:**
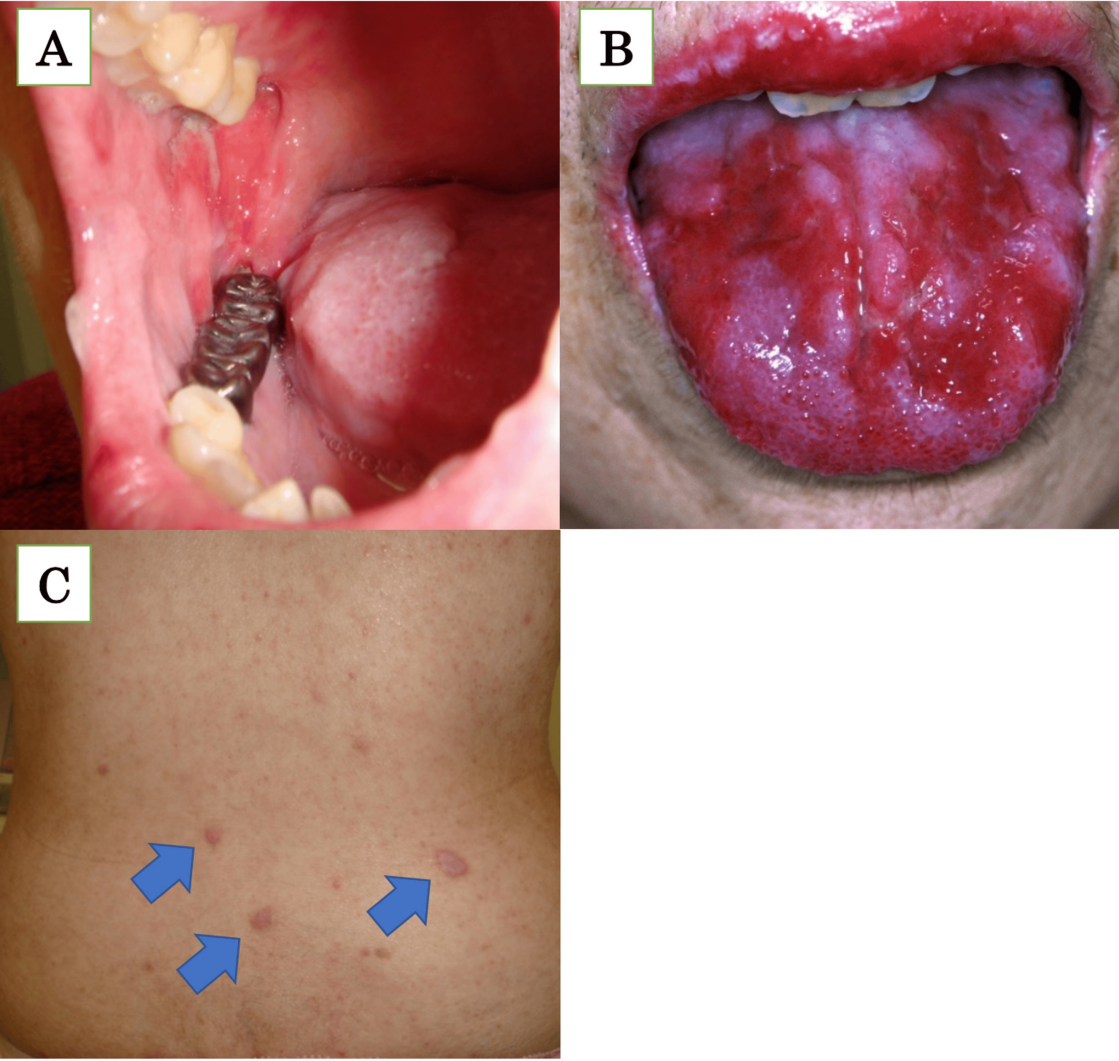
Skin findings Erosions were observed on the hard palate, buccal mucosa (A), and tongue (B). The dark red erythematous lesions with scales of approximately 1 cm were observed (C, arrow).

Two and a half years prior, the patient developed erosions and ulcers in the oral cavity and erythematous lesions on the trunk (Figure 1), which were treated with topical steroids.

**Figure 2:**
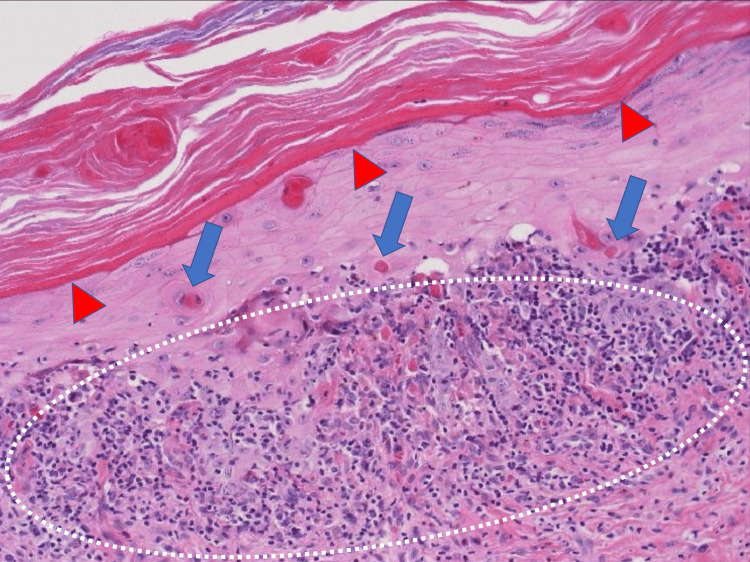
Pathological findings of the lesion in the left lower abdomen The epidermis shows hyperkeratosis and thickening of the granular cell layer (arrowhead), with numerous isolated epidermal cell necroses. In the upper dermis, there is a band of lymphocytic infiltration (circle), and at the epidermal–dermal interface, there is liquid degeneration and Civatte bodies (arrow).

The rashes did not improve, despite treatment; therefore, a skin biopsy was performed. Pathological findings of the lesion in the left lower abdomen (Figure 2) showed hyperkeratosis, thickening of the granular cell layer in the epidermis, and numerous isolated epidermal cell necroses.

In the upper dermis, there was a band of lymphocytic infiltration, and liquid degeneration and Civatte bodies were observed at the epidermal-dermal border. Based on these pathological findings, she was diagnosed with LP and was started on oral prednisolone (PSL) 20 mg/day. As LP recurred repeatedly with PSL reduction, PSL administration was maintained at a low dose.

Two years prior, she complained of breathlessness and cough and was referred to the Department of Respiratory Medicine. At the time of consultation, she had no history of smoking, dust or gas inhalation, or any respiratory illnesses. Chest auscultation revealed bilateral squawking in the lungs. Laboratory examination revealed low levels of immunoglobulins and mild elevation of C-reactive protein, while autoantibodies suggestive of rheumatoid arthritis or collagen disease were negative. With 3 L/min of oxygen, arterial blood gas analysis revealed hypoxemia (partial pressure of oxygen in the arterial blood 77.0 mmHg) and hypercapnia (partial pressure of carbon dioxide in the arterial blood 59.5 mmHg).

**Figure 3:**
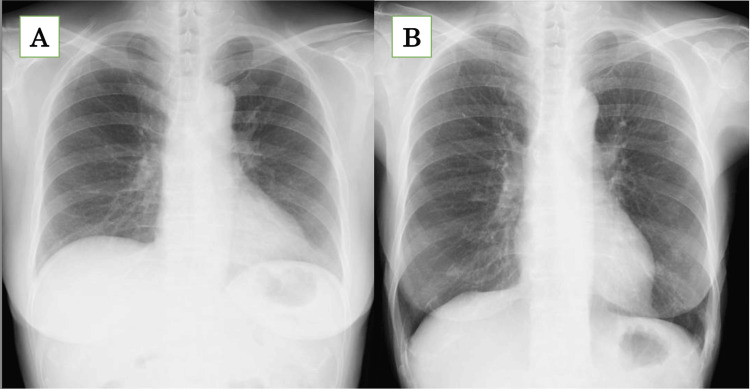
Changes over time in chest radiographs There are no abnormal findings in the lung fields, indicating that the lungs have become hyperinflated over the past four years. A: four years ago; B: just before the last recurrent malignant lymphoma

Pulmonary function tests revealed severe obstructive ventilatory impairment, with a vital capacity of 1.16 L (50.4% of the predicted value). Chest radiographs showed gradual hyperinflation of the lungs over four years (Figure 3).

Chest computed tomography (CT) revealed diffuse bronchiectasis and mild bronchial wall thickening, with no obvious mosaic pattern during the inspiratory phase and no air trapping during the expiratory phase. For chronic airway inflammation, clarithromycin (200 mg/day) was administered orally. Long-term oxygen therapy was introduced as a symptomatic therapy.

**Figure 4:**
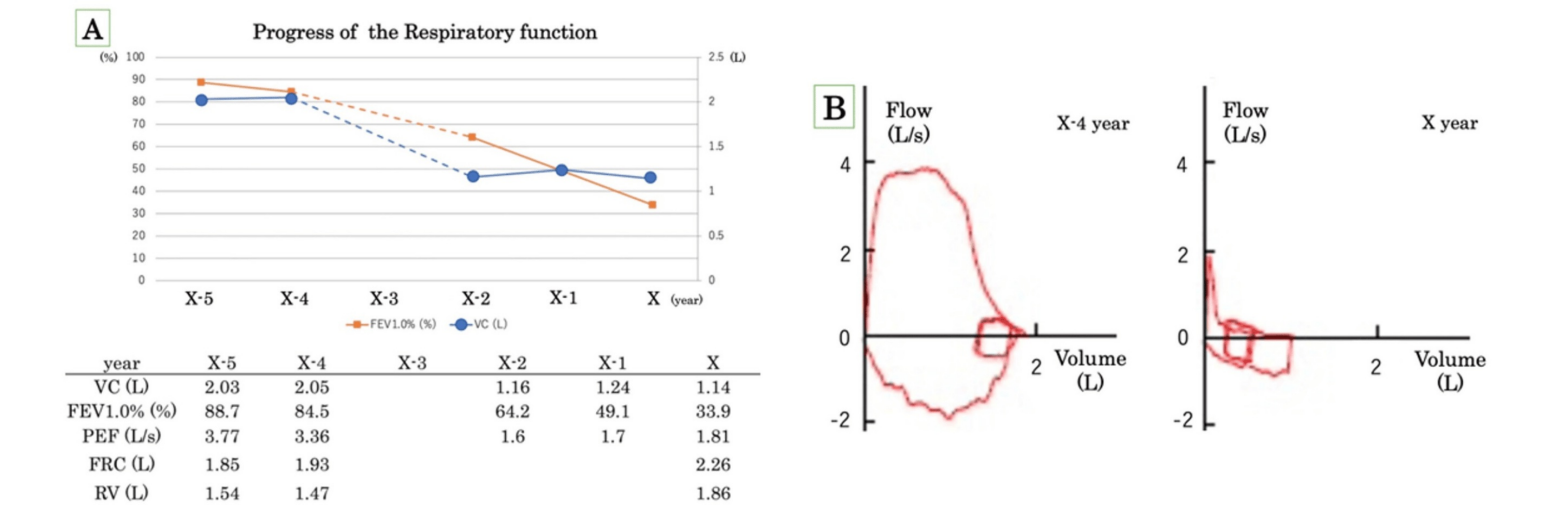
Pulmonary function test Pulmonary function tests showed a significant decrease over four years (A) and showed gradual severe mixed impairment (B). VC: vital capacity; FEV: forced expiratory volume; PEF: peak expiratory flow; FRC: functional residual capacity; RV: residual volume

Subsequently, the patient was repeatedly admitted to and discharged from the hospital because of infection and worsening respiratory distress. Her respiratory function continued to deteriorate over time (Figure 4). During this period, neither laboratory examinations nor imaging studies indicated any recurrence of lymphoma, and no chemotherapy was administered.

Finally, the patient developed dyspnea and was hospitalized because of worsening respiratory failure. Although she received antimicrobial treatment and palliative respiratory management, she died of progressive respiratory failure eight days after admission.

Autopsy findings

**Figure 5:**
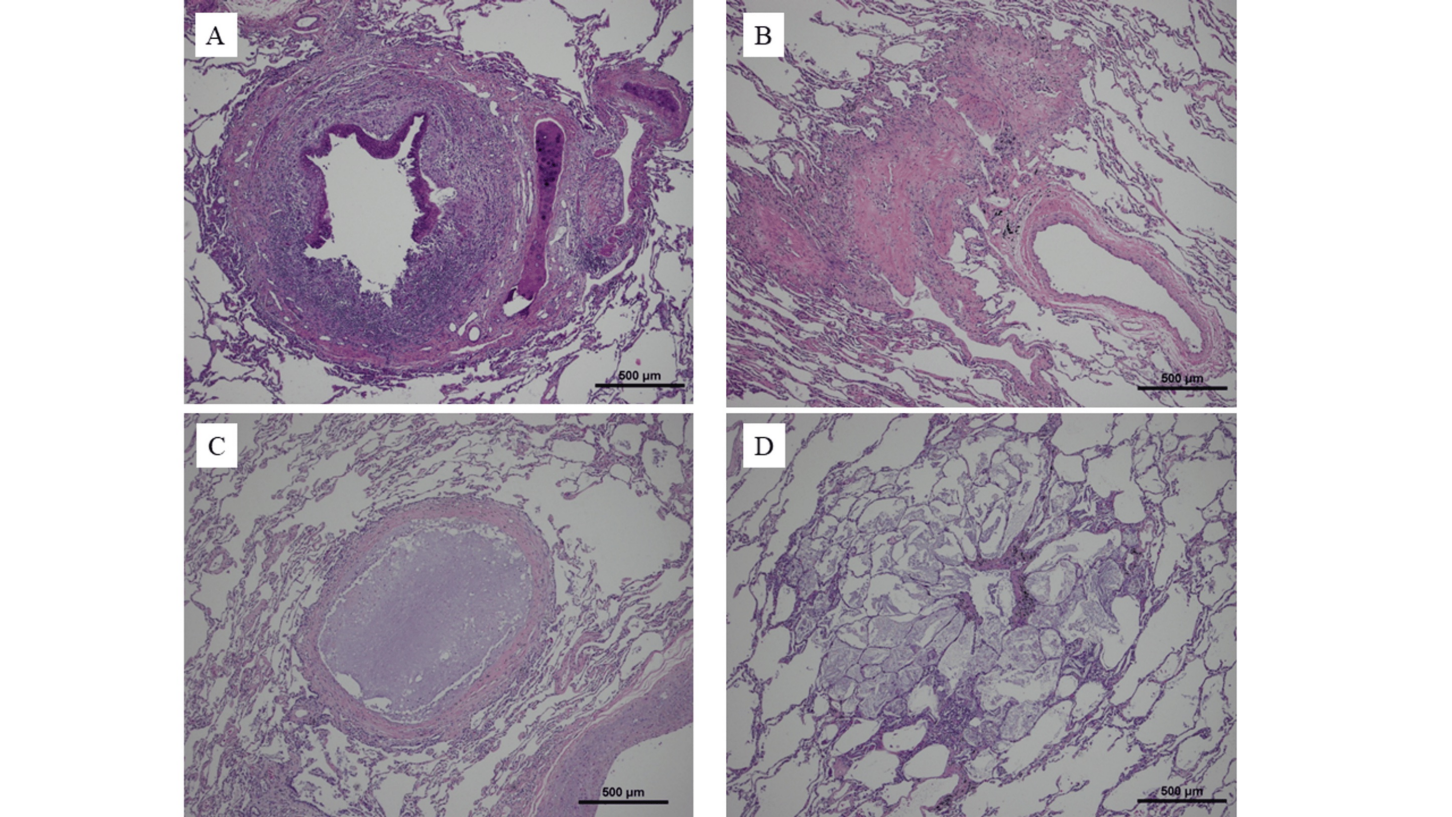
Histological findings at autopsy Occasional bronchial epithelial detachment (A), fibrosis of surrounding tissue (B), and luminal narrowing or obstruction without organizing pneumonia are seen in the bronchioles and respiratory bronchioles of the bilateral lungs (C). In the obstructed respiratory bronchial region, there is an exudate with cholesterin crystals and hyperinflation of the peripheral alveoli (D).

An autopsy showed a 5 cm-large mass on the anterior surface of the thoracic vertebrae and a 3 cm-large mass on the chest wall. Microscopic examination revealed medium-sized lymphocytes diffusely infiltrating all retrieved lymph nodes (cervical, bilateral hilar, mediastinal, and periaortic abdominal lymph nodes), the spleen, right pulmonary pleura, and bone marrow. Immunostaining revealed CD20+, CD79a+, CD3-, CD5-, CD10-, Bcl-2+, cyclin D1-, and CD30- cells. From the fine bronchi to the respiratory bronchioles of the bilateral lungs, some of the bronchial epithelium was detached, and the surrounding tissue was fibrous. The lumen was narrowed without obstruction by organic pneumonia. Some lumens were completely occluded and chordated (Figure 5).

Mild-to-moderate inflammatory cell infiltration was observed around the bronchi; however, there was no specific inflammation, such as active inflammation or epithelial granuloma, in the bronchi. There was an exudate with cholesterol crystals in the alveoli from the obstructed respiratory bronchial region. Peripheral alveoli were hyperinflated. Immunostaining revealed enlarged cells with intranuclear inclusion bodies that were positive for CMV antibodies in a small part of the middle lobe of the right lung. In addition, thickening of the epidermis and lymphocytic infiltration in the upper dermis were observed on the tongue, indicating oral LP. No other epithelial lesions were observed. Lymphocyte-like cells had infiltrated the anterior thoracic spine, right chest wall, lymph nodes, and spleen, suggesting recurrent lymphoma. The final diagnosis was BO, oral LP, recurrent B-cell lymphoma, and CMV infection. Gram and Grocott staining did not reveal the presence of any bacteria or fungi.

An antibody test was performed on skin lesions to rule out paraneoplastic pemphigus (PNP). Immunoblotting, indirect antibody methods, or enzyme-linked immunosorbent assays were performed, and all autoantibodies suggestive of other diseases, such as anti-CS antibody, anti-BMZ antibody, antibody for BP180, BP220, Dsg1/Dsg3, envoplakin, periplakin, Dsc1, Dsc2, Dsc3, type VII collagen, or laminin 332, were negative.

## Discussion

Here, we report a case of NHL complicated by steroid-dependent oral LP that developed into a fatal course due to BO. To our knowledge, this is the first report of a case of a combination of BO and LP histologically diagnosed by autopsy.

BO typically occurs in patients who have undergone lung or hematopoietic stem cell transplantation. It has been reported that up to 50% of lung transplant recipients or 2-10% of patients with allogeneic hematopoietic cell transplantation will develop BOS [3]. Other solid-organ transplantations, such as living-donor renal transplantation, can also cause BO [5]; however, non-transplant-related BO is rare. The causes of non-transplant-related BO include complications of infection, medication, inhalation of toxic gases, and autoimmune diseases such as PNP and rheumatoid arthritis.

LP is a chronic inflammatory disease of the epidermal cells [6]; it is caused by an autoimmune reaction of T cells against keratinocytes in the basal epidermis induced by antigenic stimuli, such as drugs, infections, tumors, and metals [3]. To our knowledge, only two case reports have described an association between BO and LP [7,8]. In the first report, the patient had a history of malignant fibrous histiocytoma in the retroperitoneum and developed LP during the perioperative period. The current patient died two years after surgery due to worsened respiratory failure, probably caused by BO, which was not proven by histological examination [7]. In the second report, respiratory distress and oral lesions developed simultaneously. An immunological and histological diagnosis of lichen planus was established, and a clinical diagnosis of BO was established. No malignant complications were found [8]. Both this case and the previously reported cases are consistent, as the patient died a year or two years after the onset of respiratory symptoms.

The difference between these previous reports and the present case is the presence of a coexisting disease with LP. In this case, the patient had a long history of NHL before the onset of LP, requiring differentiation from PNP, which is an autoimmune blistering disease associated with NHL. It is also known that BO occurs in approximately 20-30% of patients with PNP [9]. Both LP and PNP may be associated with tumors or manifest within the oral mucosa, making differentiation between the two conditions challenging. This difficulty is compounded by the presence of a lichenoid form of PNP. Histology, serum immunoprecipitation, and fluorescent antibody findings are important for differentiating LP from PNP [10,11]. Because no autoantibodies specific to LP have been reported, diagnosis of LP involves the exclusion of related diseases and histology [7,11]. On the other hand, autoantibodies that test positive for PNP are directed against plakin family proteins, α2‐macroglobulin‐like antigen‐I, BP180, p200 protein, desmogleins 1 and 3, and desmocollins. The sensitivity and specificity of anti-plakin antibodies for PNP are 86% and 98%, respectively [12]. All autoantibodies suggestive of other blistering diseases were also absent. Both the initial and postmortem specimens showed characteristic findings of lymphocytic infiltration in the upper dermis, fluid degeneration at the epidermal-dermal interface, and Civatte bodies, consistent with the pathological diagnosis of LP. In this case, the patient did not meet the diagnostic criteria for PNP and tested negative for autoantibodies, indicating that the skin eruption was compatible with LP.

Additionally, a case of non-paraneoplastic autoimmune subepidermal bullous disease associated with fatal BO has been reported [13]. In this case, a report was published on a patient with BO who developed an unclassified autoimmune subepidermal bullous disease, which is not classified as PNP. We would like to highlight that, in the management of patients with not only PNP but also other autoimmune diseases, it is important to consider the potential association with BO, as observed in both this case and ours.

The prognosis for patients with BO is poor, with two- to three-year survival rates after hematopoietic cell transplantation estimated at 60-75% and five-year survival rates after hematopoietic cell transplantation estimated at 40-50% [3]. Common treatments for BO include corticosteroids, rituximab, and azithromycin with fluticasone and montelukast [14-16]. Although all of these can slow the progression of BO, it is difficult to achieve remission. In this case, corticosteroids and rituximab were used to treat LP and NHL; however, they did not improve the symptoms. In refractory cases, lung allotransplantation may be considered; however, in this case, allotransplantation was not appropriate because of the patient's age and history of NHL [17]. Our patient died two years after the onset of respiratory symptoms. In addition to the progression of BO, the reasons may include relapse of the underlying NHL and CMV associated with immunodeficiency.

Reports of LP associated with BO are rare, although scattered cases of BO and PNP have been reported. Even in patients with LP and respiratory symptoms, clinicians should differentiate BO and diagnose it early.

## Conclusions

We experienced a case of fatal BO associated with LP, histologically confirmed by autopsy findings. The patient presented with concurrent NHL; however, there was no evidence of PNP despite investigations for autoantibodies, such as anti-desmoglein antibodies. As previously reported, once BO developed and progressed, the prognosis of this patient was very poor. This case indicates that various autoimmune skin diseases may be complicated by BO. BO is known to be difficult to diagnose in its early stages due to a lack of specific symptoms and imaging findings. When patients with an autoimmune skin disease complain of respiratory symptoms such as shortness of breath, the clinicians should consider conducting a pulmonary function test to evaluate for a reduction in forced expiratory volume in one second. Further, research is warranted to accumulate and analyze similar cases in the future.
